# Arbitrary Microphone Array Optimization Method Based on TDOA for Specific Localization Scenarios

**DOI:** 10.3390/s19194326

**Published:** 2019-10-07

**Authors:** Haitao Liu, Thia Kirubarajan, Qian Xiao

**Affiliations:** 1 School of Mechanotronics and Vehicle Engineering, East China Jiaotong University, Nanchang 330013, China; 2 Department of Electrical and Computer Engineering, McMaster University, Hamilton, ON L8S 4K1, Canada

**Keywords:** source localization, TDOA, array optimization, PSO

## Abstract

Various microphone array geometries (e.g., linear, circular, square, cubic, spherical, etc.) have been used to improve the positioning accuracy of sound source localization. However, whether these array structures are optimal for various specific localization scenarios is still a subject of debate. This paper addresses a microphone array optimization method for sound source localization based on TDOA (time difference of arrival). The geometric structure of the microphone array is established in parametric form. A triangulation method with TDOA was used to build the spatial sound source location model, which consists of a group of nonlinear multivariate equations. Through reasonable transformation, the nonlinear multivariate equations can be converted to a group of linear equations that can be approximately solved by the weighted least square method. Then, an optimization model based on particle swarm optimization (PSO) algorithm was constructed to optimize the geometric parameters of the microphone array under different localization scenarios combined with the spatial sound source localization model. In the optimization model, a reasonable fitness evaluation function is established which can comprehensively consider the positioning accuracy and robustness of the microphone array. In order to verify the array optimization method, two specific localization scenarios and two array optimization strategies for each localization scenario were constructed. The optimal array structure parameters were obtained through numerical iteration simulation. The localization performance of the optimal array structures obtained by the method proposed in this paper was compared with the optimal structures proposed in the literature as well as with random array structures. The simulation results show that the optimized array structure gave better positioning accuracy and robustness under both specific localization scenarios. The optimization model proposed could solve the problem of array geometric structure design based on TDOA and could achieve the customization of microphone array structures under different specific localization scenarios.

## 1. Introduction

In the past two decades, microphone array technology has consistently been a hot research field. Microphone arrays are mainly used for sound source localization and identification, and have been an important practical technology with many valuable applications, such as noise source localization [1,2], target sound source tracking [3], teleconferencing systems [4,5], intelligent robots [6,7,8], and so on.

In microphone array technology, there are three main methods for sound source localization, namely, beamforming, acoustic holography, and time difference of arrival (TDOA). The beamforming method applies delay-and-sum to signals from an array of microphones, and in the direction of the source, a beam peak will form to locate the sound sources [9]. Acoustic holography reconstructs the acoustic fields to locate the sound sources by solving the inverse propagation problems [10]. Beamforming and acoustic holography methods usually involve planar microphone arrays and calculation points located on a surface at a certain distance with respect to the array, which provides a poor resolution in the direction perpendicular to the array. In recent years, beamforming with several deconvolution techniques [11] and inverse methods with additional issues [12,13] have been proposed to construct volumetric sound source imaging, which can give the exact three-dimensional (3D) coordinates of sound sources. The method based on TDOA, virtually a triangulation method, locates the sound source using geometric relationships between microphones and sound sources, which can give the spatial position of sound sources with reasonable accuracy using a small number of sensors [14]. TDOA methods have been widely used for real-time sound source localization [15,16]. Moreover, in some sound source localization scenarios, such as simple sound source tracking, TDOA methods show better application prospects.

Different numbers of microphones and different kinds of array structures are used in these three methods. In general, the number of microphones used in beamforming and acoustic holography is much larger than in the TDOA method because the number of microphones has a significant influence on the reconstruction accuracy of the sound source mapping [17]. Nevertheless, the number of microphones required in the TDOA method is much smaller. In theory, only four microphones are needed to locate a sound source in three-dimensional space. For example, Wu and Zhu [15] used only four microphones to locate arbitrarily time-dependent acoustic sources in a free three-dimensional space in real-time. In addition, the number of microphones is not the decisive factor of its location accuracy. The array structure is another main factor that relates to the accuracy of source localization for these three methods.

Many kinds of microphone array structures are applied in sound source localization, which can mainly be divided into three categories: 1-dimensional, 2-dimensional, and 3-dimensional. The 1-dimensional array structure group mainly comprises binaural arrays [18] and linear arrays [19]. 2-dimensional array structures include square [20], cross [21], spiral [22,23], and circular geometries [24]. 3-dimensional arrays mainly include cubic [25], pyramidal [25], hemispherical [26], and spherical [27] geometries. Relevant scholars have analyzed the performance of various arrays, indicating that each kind of array structure is only suitable for specific localization algorithms and scenarios. There is no array structure that can achieve good localization performance under any kinds of scenarios and algorithms. For example, the two- or three- dimensional localization accuracy of a randomly distributed array will vary widely with respect to the relative position of the sound source [28]. Therefore, the optimization of the microphone array structure becomes an important research point. Wang and Bei [29] proposed an optimization method based on acoustic holography theory to optimize the microphone array coordinates on a fixed cross X-type array structure, and the main side lobe ratio and the main lobe area were selected as the optimization objective function. Kodrasi et al. [30] adopted different heuristic optimization approaches and an exhaustive search approach to optimize the microphone positions for an arbitrary planar array based on the beamforming method, and Kodrasi’s methods found near-optimal configurations. Recently, Yan and Ma [31], Sarradj [32], Bjelić et al. [33], Teng and Lv [34], and Le Courtois et al. [35] also proposed new methods for planar array optimization based on the beamforming method, and compared the array performance under different localization scenarios. In the optimization procedure, the main-lobe width and side-lobe level are generally selected as the optimization objective function. Padois et al. [36] proposed a spherical microphone array with polyhedral discretization and compared it with a spherical array with a slightly different geometry based on the beamforming method. The results showed that the polyhedral discretization array could obtain better positioning accuracy. In 2019, Padois et al. [37] carried out further research on array geometry optimization based on time-domain beamforming. They proposed an optimal spherical microphone array geometry using a nonlinear optimization. Numerical and experimental results showed that the optimized geometry improved the sound source mapping.

From the above, it can be seen that a great deal of research work has been done in the field of microphone array optimization for sound source localization. However, these array optimization methods are mainly based on the beamforming and acoustic holography methods. The optimization procedure is also usually based on existing array structures, such as cross array [38], circle array [31], spiral array [32], irregular planar array [33,34], spherical array [38], and so on. As such, certain constraints for the array structure have been introduced to the optimization. A pre-constrained array structure may lead to a local optimum, which may not be suitable for certain specific localization scenarios. In addition, besides the array optimization based on beamforming and acoustic holography, the research on array optimization based on TDOA is relatively rare. Zietlow et al. [39] established a simulation model based on TDOA to compare the source positioning accuracy of different microphone arrangements. The microphone arrays consist of eight microphones in three different arrangements, namely cube, twisted cube, and random. These array arrangements were fixed, and no optimization was performed for the array structures. Hu et al. [40] proposed an analytical method based on TDOA to optimize microphone array structure, which could guarantee that the sound source localization had the same performance in all directions for omni-directional estimation. However, the optimal result led to a set of nonlinear equations which could not give deterministic analytical solutions. With additional constraints, only a particular solution in a regular polyhedron form can be obtained. Further, only five kinds of array structures with a specified number of microphones belong to the solution of regular polyhedron form, including the tetrahedron (5 microphones), the hexahedron (9 microphones), the octahedron (7 microphones), the dodecahedron (21 microphones), and the icosahedron (13 microphones). The limited solution of array structures restricts the practical application of Hu’s method. Meanwhile, due to some constraints in modeling and solving the method, these five kinds of array structures may not give the best positioning results under some specific localization scenarios, such as the scenarios with the asymmetrical distribution of sound sources. Therefore, more in-depth research needs to be carried out in the field of array structure optimization for sound source localization based on TDOA.

This paper is devoted to an arbitrary microphone arrays optimization method for sound source localization based on TDOA. The method proposed is a numerical approach based on the particle swarm optimization (PSO) algorithm, which can optimize the array structure of an arbitrary number of microphones under any specific localization scenarios without prior array structure information. Examples of localization scenarios were constructed to obtain the optimal array structures through the proposed method. Additionally the optimal array structures were compared with the array structures proposed by Hu et al. [40] as well as random array structures under the constructed specific scenarios.

This article makes four main contributions. First, a numerical approach of microphone array optimization based on the PSO algorithm for the TDOA method is proposed. Second, the proposed model can perform array structure optimization with an arbitrary number of microphones, and no prior array structure information is introduced into the optimization procedures, which is likely to obtain the more optimal solutions. Third, the array optimization model has general applicability, which can effectively solve the problem of microphone arrangements in sound source localization under different specific localization scenarios. Fourth, the fitness evaluation function constructed in the optimization model can give good consideration to the accuracy and robustness of sound source localization based on TDOA. The two specific localization scenarios established here verify the proposed optimization method.

In the following sections, the optimization model is introduced in detail, and the localization performance is compared with the array structures proposed by Hu et al. as well as with random array structures. Section 2 introduces the construction of the TDOA-based sound source localization model for an arbitrary microphone array, as well as the solution for the localization model. The numerical optimization model based on PSO for an arbitrary array structure is presented in Section 3, in addition to the optimization procedure. Simulations were performed and their results are discussed in Section 4, followed by conclusions in Section 5.

## 2. Construction of Localization Model Based on TDOA

The sound source localization model is the basis of the array structure optimization. The TDOA method was used to locate the sound source. Therefore, the time difference and the spatial geometric relationship between the array and the sound source were used to establish the localization model.

### 2.1. Geometric Structure Parameterization for Arbitrary Microphone Array

**Figure 1:**
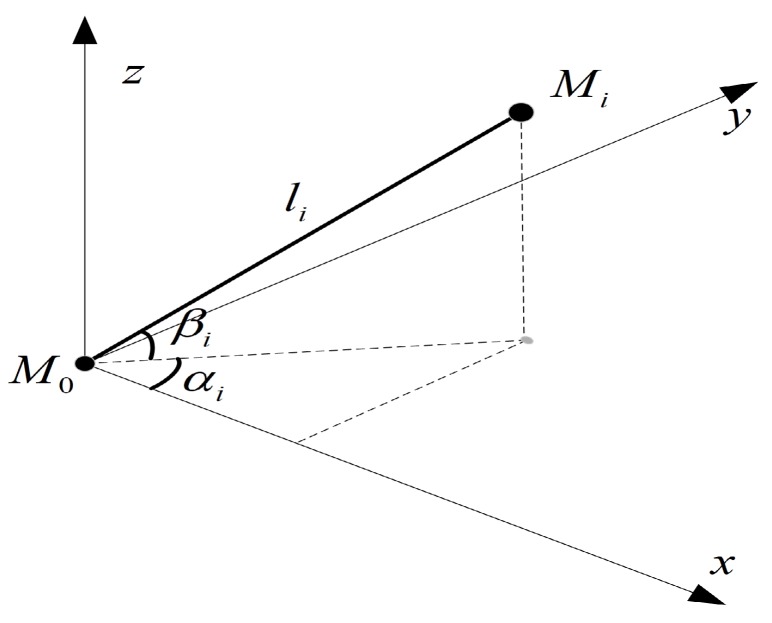
Diagram of the microphones’ coordinates.

In order to optimize the microphone array, the geometric structure of the array needs to be parameterized first. Because the sound source localization method in this paper is based on TDOA, a reference microphone is needed in the microphone array. For convenience, the coordinate of the reference microphone $M_{0}$ is set as (0,0,0). Then, the other microphones’ coordinates can be expressed by the radial distance $l_{i}$, the azimuth angle $\alpha_{i}$, and the elevation angle $\beta_{i}$ in three-dimensional space, as shown as Figure 1.

The coordinates of the other microphones are $M_{i}\left(l_{i}\cos\left(\beta_{i}\right)\cos\left(\alpha_{i}\right),l_{i}\cos\left(\beta_{i}\right)\sin\left(\alpha_{i}\right),l_{i}\sin\left(\beta_{i}\right)\right)$, where $i=1,2,3...,N_{m}$, $N_{m}$ stands for the number of microphones except for the reference microphone. Therefore, the optimal parameter of the microphone array is

(1) $$M_{p}=\left(\begin{array}{ccc} l_{1} & \alpha_{1} & \beta_{1}\\ l_{2} & \alpha_{2} & \beta_{2}\\ \vdots & \vdots & \vdots \\ l_{i} & \alpha_{i} & \beta_{i} \end{array}\right)\;\;\;\;\;\;i=1,2,\cdots ,N_{m}.$$

The constraint of the azimuth angle $\alpha_{i}$ is $\left[0^{\circ },360^{\circ }\right]$ and for the elevation angle it is $\beta_{i}$ is $\left[-90^{\circ },90^{\circ }\right]$. The range of radial distance $l_{i}$ is related to the size of microphone array and the frequency band of sound source, as well as the requirements for actual positioning scenarios. Normally, the lower limit of $l_{i}$ is the diameter of microphone $d_{m}$, and the upper limit of $l_{i}$ is $c/(2f_{m})$, where *c* is the speed of sound and $f_{m}$ is the main periodic frequency present in the sources. Then, the set of the optimal search space can be described as

(2) $$\begin{array}{c} \mathrm{OS}=\{M_{p}|M_{p}=\left(\begin{array}{ccc} l_{1} & \alpha_{1} & \beta_{1}\\ \vdots & \vdots & \vdots \\ l_{i} & \alpha_{i} & \beta_{i} \end{array}\right),l_{i}\in \left[d_{m},c/2f_{m}\right],\alpha_{i},\beta_{i}\in \left[0^{\circ },360^{\circ }\right],i=1,2,\cdots ,N_{m}\}. \end{array}$$

### 2.2. Spatial Source Localization Model Based on TDOA

The spatial source location model was constructed based on the TDOA method, which is a triangulation method. Suppose the coordinate of the sound source is S=(x,y,z). The mathematical description is shown as follows:

(3) $$\left\{\begin{array}{cc} r_{0} & =\sqrt{{\left(x_{0}-x\right)}^{2}+{\left(y_{0}-y\right)}^{2}+{\left(z_{0}-z\right)}^{2}}=\left|\right|M_{0}-S\left|\right|,\\ r_{i} & =\sqrt{{\left(x_{i}-x\right)}^{2}+{\left(y_{i}-y\right)}^{2}+{\left(z_{i}-z\right)}^{2}}=\left|\right|M_{i}-S\left|\right|, \end{array}\right.$$

where $r_{0}$ is the distance between the sound source and the reference microphone $M_{0}$, and $r_{i}$ is the distance between the sound source and the other microphones $M_{i}$. $i=1,2,\cdots ,N_{m}$.

By constructing the time arrival difference from the sound source to the reference microphone and the other microphones, the spatial source location model can be obtained. The model is shown as follows:

(4) $$r_{i,0}=\left|\right|M_{i}-S||-\left|\right|M_{0}-S\left|\right|=c\tau_{i,0},$$

where $\tau_{i,0}$ is the sound arrival time difference between $M_{i}$ and $M_{0}$. $\tau_{i,0}$ can be estimated by the method of cross correlation.

Suppose that $u_{i}\left(t\right)$ and $u_{0}\left(t\right)$ are the acoustic signals acquired by microphones $M_{i}$ and $M_{0}$ separately. The cross-correlation function between the two signals is

(5) $$R_{i,0}\left(\tau \right)=\int_{-\infty }^{\infty }u_{i}\left(t\right)u_{0}\left(t+\tau \right)dt,$$

(6) $$\tau_{i,0}=\arg\;\max\left[R_{i,0}\left(\tau \right)\right].$$

The spatial source location model consists of a group of nonlinear multivariate equations, which is difficult to solve. An alternative method is to transform the model into a set of linear equations. Spatial distance satisfies the relationship shown in Equation (7):

(7) $$r_{i}^{2}={\left(r_{i,0}+r_{0}\right)}^{2}.$$

Then, Equation (4) can be rewritten as:

(8) $$2r_{i,0}r_{0}+2x_{i,0}x+2y_{i,0}y+2z_{i,0}z=K_{i}-K_{0}-r_{i,0}^{2},$$

where $K_{i}=x_{i}^{2}+y_{i}^{2}+z_{i}^{2}$, $K_{0}=x_{0}^{2}+y_{0}^{2}+z_{0}^{2}$, $x_{i,0}=x_{i}-x_{0}$, $y_{i,0}=y_{i}-y_{0}$, $z_{i,0}=z_{i}-z_{0}$.

Equation (8) is a group of linear equations, which can be written in matrix form:

(9) AX=B

where $X={\left[x,y,z\right]}^{T}$, $A=-\left(\begin{array}{ccc} x_{i,0} & y_{i,0} & z_{i,0}\\ \vdots & \vdots & \vdots \\ x_{N,0} & y_{N,0} & z_{N,0} \end{array}\right)$, $B=\left(\begin{array}{c} r_{i,0}\\ \vdots \\ r_{N,0} \end{array}\right)\cdot r_{0}+1/2\left(\begin{array}{c} r_{i,0}^{2}-K_{1}+K_{0}\\ \vdots \\ r_{N,0}^{2}-K_{N}+K_{0} \end{array}\right)$.

### 2.3. Solution for Spatial Source Localization model

When the number of microphones is $N_{m}=3$, the spatial source location model (Equation (9)) can be solved directly, as represented in [41]. However, the direct solution method may produce two different answers, which leads to localization ambiguity. Meanwhile, the accuracy and robustness of source localization are not very good under the condition of $N_{m}=3$. Adding redundant sensors can effectively improve the performance of source localization. When there are more than four microphones ($N_{m}\geq 4$), the system is overdetermined, as the number of measurements is greater than the number of unknowns. The LS (least-square) method can be used to solve the overdetermined linear equations. Chan and Ho [42] proposed an alternative solution algorithm in closed-form, valid for both distant and close sources, which used twice-weighted LS to give the localization results. Chan’s method gives an explicit solution with reasonable accuracy and is non-iterative with low computational complexity. Therefore, Chan’s method is more suitable for the optimization calculation of acoustic array structure in this paper.

#### 2.3.1. The First Weighted Least-Square Solution Process

In order to solve the source localization model by least-square method, Equation (9) should be rewritten to construct an error vector. Because of noise in the TDOA estimation, the error vector can be derived as:

(10) $$\psi =h-G_{a}z_{a}^{0},$$

where $z_{a}={\left[x,y,z,r_{0}\right]}^{T}$ is the unknown vector. $h=1/2\left(\begin{array}{c} r_{1,0}^{2}-K_{1}+K_{0}\\ \vdots \\ r_{N,0}^{2}-K_{N}+K_{0} \end{array}\right)$. $G_{a}=-\left(\begin{array}{cccc} x_{1,0} & y_{1,0} & z_{1,0} & r_{1,0}\\ \vdots & \vdots & \vdots & \vdots \\ x_{N,0} & y_{N,0} & z_{N,0} & r_{i,0} \end{array}\right)$. ${\left(\cdot \right)}^{2}$ stands for the expectations of variables without noise.

Suppose that the noise of TDOA estimation is $n_{i}$. $\tau_{i,0}=\tau_{i,0}^{2}+n_{i,0}$. $r_{i,0}=r_{i,0}^{0}+cn_{i,0}$, and $r_{i}^{0}=r_{i,0}^{0}+r_{0}^{0}$. Then, ψ can be expressed as

(11) $$\psi =cRn+0.5c^{2}n⊙n,$$

where $R=\mathrm{diag}r_{1}^{0},r_{2}^{0},\cdots ,r_{N}^{0}$, $n={\left[n_{1,0},n_{2,0},\cdots ,n_{N,0}\right]}^{T}$. The symbol ⊙ stands for the Schur product. The noise vector n approximately obeys a Gaussian normal distribution.

In practice, the condition $r_{i}^{0}\gg r_{i,0}=cn_{i,0}$ is usually satisfied. Therefore, the second term on the right hand side of Equation (11) can be ignored. Then, the covariance matrix of ψ can be given as:

(12) $$\Psi =E\left[\psi \psi^{T}\right]=c^{2}RQR,$$

where $Q=E\left(nn^{T}\right)=\mathrm{Cov}\left(n\right)$. Then, the first weighted LS method is used to solve Equation (10):

(13) $$z_{a}={\left(G_{a}^{T}\Psi^{-1}G_{a}\right)}^{-1}G_{a}^{T}\Psi^{-1}h.$$

When the source is far from the array, each $r_{i}^{0}$ is close to $r^{0}$, so $R\approx r^{0}I$. Then, an approximate solution of Equation (13) is

(14) $$z_{a}\approx {\left(G_{a}^{T}Q^{-1}G_{a}\right)}^{-1}G_{a}^{T}Q^{-1}h.$$

When the source is close to the array, Equation (14) can be firstly used to obtain an initial solution to estimate R, which can be substituted into Equations (12) and (13) to get a more accurate result.

#### 2.3.2. The Second Weighted Least-Square Solution Process

The above solution of $z_{a}$ assumes that *x*, *y*, and $r_{0}$ are independent. However, $r_{0}$ is related to the source location. In order to incorporate this relationship to give an improved estimate, $z_{a}$ can be expressed as

(15) $$z_{a,1}=x^{0}+e_{1},z_{a,2}=y^{0}+e_{2},z_{a,3}=z^{0}+e_{3},z_{a,4}=r_{0}^{0}+e_{4},$$

where $e_{1}$, $e_{2}$, $e_{3}$, and $e_{4}$ are estimation errors of $z_{a}$. $\left(x^{0},y^{0},z^{0}\right)$ are the coordinates of the real sources. Then, a new error vector $\psi^{\prime }$ can be obtained as:

(16) $$\psi^{\prime }=h^{\prime }-G_{a}^{\prime }z_{a}^{\prime },$$

where $h^{\prime }=1/2\left(\begin{array}{c} {\left(z_{a,1}-x_{0}\right)}^{2}\\ {\left(z_{a,2}-y_{0}\right)}^{2}\\ {\left(z_{a,3}-z_{0}\right)}^{2}\\ z_{a,4}^{2} \end{array}\right)$, $G_{a}^{\prime }=\left(\begin{array}{ccc} 1 & 0 & 0\\ 0 & 1 & 0\\ 0 & 0 & 1\\ 1 & 1 & 1 \end{array}\right)$, $z_{a}^{\prime }=\left(\begin{array}{c} {\left(x-x_{1}\right)}^{2}\\ {\left(y-y_{1}\right)}^{2}\\ {\left(z-z_{1}\right)}^{2} \end{array}\right)$.

Substitute Equation (15) into Equation (16):

(17) $$\psi^{\prime }=\left(\begin{array}{c} 2\left(x^{0}-x_{0}\right)e_{1}+e_{1}^{2}\\ 2\left(y^{0}-y_{0}\right)e_{2}+e_{2}^{2}\\ 2\left(z^{0}-z_{0}\right)e_{3}+e_{3}^{2}\\ 2r_{0}^{0}e_{4}+e_{4}^{2} \end{array}\right)\approx \left(\begin{array}{c} 2\left(x^{0}-x_{0}\right)e_{1}\\ 2\left(y^{0}-y_{0}\right)e_{2}\\ 2\left(x^{0}-z_{0}\right)e_{3}\\ 2r_{0}^{0}e_{4}+e_{4}^{2} \end{array}\right).$$

The covariance matrix of $\psi^{\prime }$ is

(18) $$\Psi^{\prime }=E\left[\psi^{\prime }\psi^{\prime T}\right]=4R^{\prime }\mathrm{cov}\left(z_{a}\right)R^{\prime },$$

where $R^{\prime }=\mathrm{diag}\left\{x^{0}-x_{0},y^{0}-y_{0},z^{0}-z_{0},r_{0}^{0}\right\}$. Then, the second weighted LS method is used to solve Equation (16):

(19) $$z_{a}^{\prime }={\left(G_{a}^{\prime T}\Psi^{\prime -1}G_{a}^{\prime }\right)}^{-1}G_{a}^{\prime T}\Psi^{\prime -1}h^{\prime }.$$

The matrix $\Psi^{\prime }$ is not known since it contains the true values. However, $R^{\prime }$ can be approximated by using the values in $z_{a}$. If the source is far away, then the covariance matrix of $z_{a}$ can be represented as:

(20) $$\mathrm{cov}\left(z_{a}\right)\approx c^{2}{r^{0}}^{2}{\left(G_{a}^{T}Q^{-1}G_{a}\right)}^{-1}.$$

Then, Equation (19) reduces to:

(21) $$\begin{array}{c} z_{a}^{\prime }\approx {\left(G_{a}^{\prime T}R^{\prime -1}G_{a}^{T}Q^{-1}G_{a}R^{\prime -1}G_{a}^{\prime }\right)}^{-1}\left(G_{a}^{\prime T}R^{\prime -1}G_{a}^{T}Q^{-1}G_{a}R^{\prime -1}\right)h^{\prime } \end{array}.$$

The final sound source position is estimated as:

(22) $$z_{p}=\sqrt{z_{a}^{\prime }}+{\left[x_{0},y_{0},z_{0}\right]}^{T}$$

or

(23) $$z_{p}=-\sqrt{z_{a}^{\prime }}+{\left[x_{0},y_{0},z_{0}\right]}^{T}.$$

## 3. Numerical Optimization Method for Array Structures

Given a certain number of microphones, there are infinite spatial geometric structures for microphone arrays. Nevertheless, in various practical scenarios, it is necessary to find the optimal array structure to effectively reduce the positioning error of sound sources in the target area. Because the microphone array consists of multiple microphones and each microphone’s coordinates have three independent variables, it the structure optimization of the microphone array in this paper is a multidimensional optimization problem.

The evolutionary algorithm is a global optimization method with high robustness and broad applicability. Unlike classic optimization methods such as gradient descent and quasi-Newton methods, the gradient of the problem being optimized is not required for the evolutionary algorithm. Meanwhile, the evolutionary algorithm makes few or no assumptions about the optimization problem and has great advantages in the application of unsupervised, complex multidimensional problems that cannot be solved using traditional deterministic algorithms [43]. The genetic algorithm (GA) and particle swarm optimization (PSO) algorithm are evolutionary algorithms. GA searches for the optimal solution by imitating the mechanism of selection and inheritance in nature. The selection of crossover rate and mutation rate in GA seriously affects the quality of the solution, and the selection mostly depends on experience. Additionally, GA is very slow and difficult to converge for high-dimensional problems. Particle swarm optimization (PSO) is a metaheuristic global optimization algorithm, and the inner workings of the PSO make sufficient use of probabilistic transition rules to search very large spaces of candidate solutions in parallell [44]. Compared with GA, PSO has the advantage of simplicity, easy implementation, and few parameters requiring adjustment. PSO does not have genetic operations such as crossover and mutation. Instead, it determines the search based on its speed. Another essential feature of PSO is that particles have memories. The full search and update process of PSO follows the current optimal solution. Compared with GA, PSO may converge to an optimal solution more quickly. For the optimization problem of the microphone array structure in this paper, the gradient of the optimization objective function is difficult to derive. Additionally, the structure optimization of an array with many microphones is a high-dimensional optimization problem. These factors make PSO an effective method to solve the optimization problem in this paper. Therefore, an optimization model based on PSO was constructed to optimize the geometric parameter of the microphone array under different localization scenarios.

### 3.1. Optimization Model Based on PSO

A swarm of particles which traverse a multidimensional search space are employed in the PSO algorithm to find optima. Each particle is a potential solution and is influenced by the experiences of other particles, as well as its own experiences. Let $p_{j}$ be the position in the search space of the *j*-th particle, and the number of particles is set as $N_{p}$. Then, a swarm of particles can be expressed as:

(24) $$P_{s}=\left[p_{1},p_{2},\cdots ,p_{N_{P}}\right],$$

where each particle can be denoted as

(25) $$p_{j}=\left(\begin{array}{ccc} l_{1} & \alpha_{1} & \beta_{1}\\ l_{2} & \alpha_{2} & \beta_{2}\\ \vdots & \vdots & \vdots \\ l_{N_{m}} & \alpha_{N_{m}} & \beta_{N_{m}} \end{array}\right)\;\;\;\;\;j=1,2,\cdots ,N_{p}.$$

A new fitness evaluation function for the array structure optimization is constructed by the mean squared error (MSE) and the variance (VAR) of the localization results, which can comprehensively consider the localization accuracy and robustness. The fitness function is shown as follows:

(26) $$f\left(p_{j}\right)=\phi_{w}\mathrm{MSE}\left(z_{p}\right)+\left(1-\phi_{w}\right)\mathrm{VAR}(z_{p}),$$

where $\phi_{w}$ is the weight value, $\phi_{w}\in \left[0,1\right]$. $z_{p}$ is the final estimated sound source position. $\mathrm{MSE}(z_{p})$ is the mean squared error of the localization results, which can be defined as

(27) $$\mathrm{MSE}\left(z_{p}\right)=\frac{1}{N_{s}}\sum_{i}^{N_{s}}{∥z_{pi}-z_{pi}^{0}∥}^{2},$$

where $z_{p}^{0}$ is the coordinate of the real source. $N_{s}$ is the number of sources involved in the optimization. $\mathrm{VAR}(z_{p})$ is the variance of the localization results, which can be defined as:

(28) $$\mathrm{VAR}\left(z_{p}\right)=\frac{1}{N_{s}}\sum_{i}^{N_{s}}{\left(∥z_{pi}-z_{pi}^{0}∥-\bar{∥z_{pi}-z_{pi}^{0}∥}\right)}^{2},$$

where $\bar{∥z_{p}-z_{p}^{0}∥}=\frac{1}{N_{s}}\sum_{i}^{N_{s}}∥z_{pi}-z_{pi}^{0}∥$.

In Equation (26), the mean squared error can be used to judge the accuracy of sound source location results, and the variance can be used to judge the robustness of sound source localization results. The weight ratio between them can be adjusted according to the requirement of localization scenarios.

Then, an optimization problem (minimization) is defined as:

(29) $$\begin{array}{c} \exists p_{j}^{\prime }\in \mathrm{OS}\subseteq R^{d}\Rightarrow \forall p_{j}\in \mathrm{OS},f\left(p_{j}^{\prime }\right)\leq f\left(p_{j}\right)\;\;\;\;\;\;j=1,2,\cdots ,N_{p}, \end{array}$$

where $R^{d}$ is the real number field in *d*-dimensional space.

The PSO algorithm is used to solve this optimization problem. To seek the optimal solution, each particle moves in the direction of its previously best ($p_{best}$) position and the global best ($g_{best}$) position in the swarm. The expression of $p_{best}$ is

(30) $$\begin{array}{c} p_{best}\left(j,k\right)=\arg\;\min\left[f\left(p_{j}\left(k\right)\right)\right]\;\;\;\;\;j\in \left\{1,2,\cdots ,N_{p}\right\};k=1,\cdots ,I_{t}, \end{array}$$

and the expression of $g_{best}$ is

(31) $$\begin{array}{c} g_{best}\left(k\right)=\arg\;\min\left[f\left(p_{j}\left(k\right)\right)\right]\;\;\;\;\;\;j=1,2,\cdots ,N_{p};k=1,\cdots ,I_{t}, \end{array}$$

where *k* denotes the current iteration number, and $I_{t}$ denotes the maximum iteration number.

The velocity V and position p of particles are updated by the following equations:

(32) $$\begin{array}{c} V_{j}\left(k+1\right)=wV_{j}\left(k\right)+c_{1}\mathrm{rand}\left(\cdot \right)\left(p_{best}\left(j,k\right)-p_{j}\left(k\right)\right)+c_{2}\mathrm{rand}\left(\cdot \right)\left(g_{best}\left(j,k\right)-p_{j}\left(k\right)\right), \end{array}$$

(33) $$p_{j}\left(k+1\right)=p_{j}\left(k\right)+V_{j}\left(k+1\right),$$

where V stands for the migration velocity of particles, which is common to be set as a boundary to limit particles flying out of the search space. rand(·) are uniformly distributed random variables within range [0,1]. $c_{1}$ and $c_{2}$ stand for learning factors, which are positive constant parameters. *w* is the inertia weight used to balance the global exploration and local exploitation. Shi [45] suggested a solution to determine the inertia weight:

(34) $$w_{t}=w_{max}-\frac{w_{max}-w_{min}}{I_{t}}k,$$

where $w_{max}$ and $w_{min}$ are maximum and minimum weight, respectively.

### 3.2. PSO Optimization Procedure

The optimization procedure for the acoustic array is summarized as follows:

Step 1. Initialize PSO parameters including the number of particles $N_{p}$, the learning factors $c_{1}$ and $c_{2}$, inertia weights $w_{max}$ and $w_{min}$, and the total iteration number $I_{t}$.

Step 2. Initialize the particles’ positions with a random distribution $p_{j}\left(0\right)$, and the parameters of the each particle ($j=1,2,\ldots ,N_{p}$) do not go beyond the boundaries of the search space.

Step 3. Initialize $p_{best}\left(j,0\right)=p_{j}\left(0\right)$; $g_{best}\left(0\right)=\mathrm{argmin}f\left(p_{j}\left(0\right)\right)$.

Step 4. Update particles’ velocity and position through Equations (30) and (31), and maintain $p_{j}\left(k+1\right)\in \mathrm{OS}$.

Step 5. If $f\left(p_{j}\left(k\right)\right)<f\left(p_{best}\left(j,k\right)\right)$, update the best known particle position $p_{best}\left(j,k\right)=p_{j}\left(k\right)$; if $f\left(p_{best}\left(j,k\right)\right)<f\left(g_{best}\left(k\right)\right)$, update the global best position $g_{best}\left(k\right)=p_{best}\left(j,k\right)$.

Step 6. Judge the termination criteria: $f(g_{best}\left(k\right))\leq \delta$ (δ is presented as threshold) or the iteration number reaches the maximum $I_{t}$ with the fitness function converging steadily. If not, repeat Steps 4 and 5. Otherwise, go to Step 7.

Step 7. Output the $g_{best}\left(k\right)$ that stands for the best optimized result.

**Figure 2:**
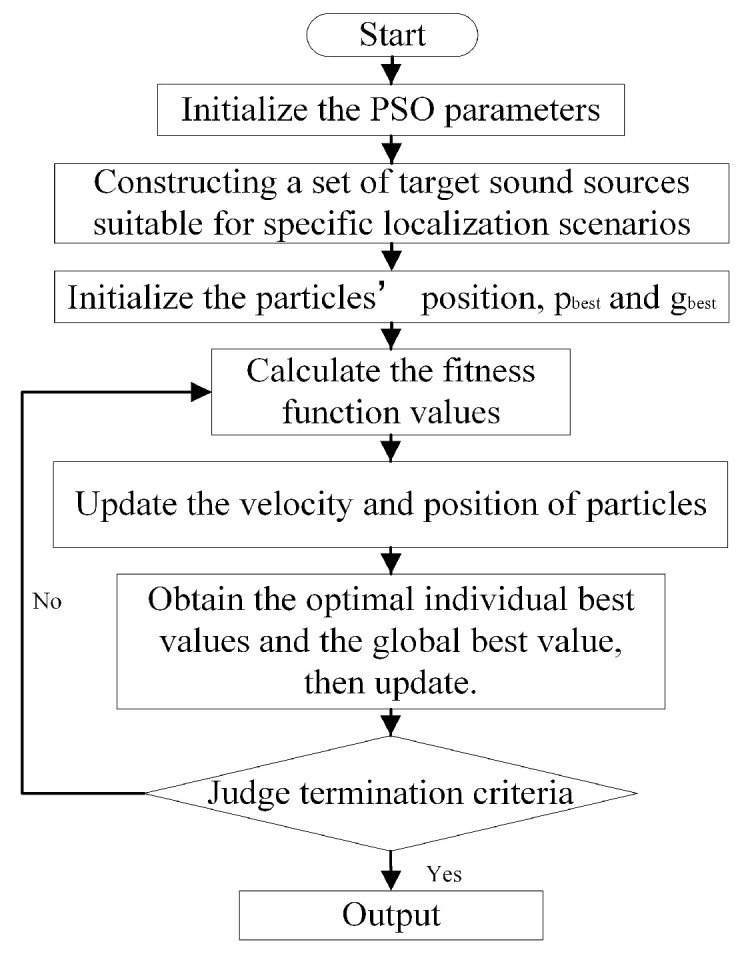
Flow chart for microphone array optimization. PSO: particle swarm optimization.

The flow chart of the optimization procedure is shown in Figure 2.

## 4. Simulation and Analysis

In order to verify the effectiveness of the method proposed in this paper, two kinds of sound source localization scenarios were constructed for microphone array optimization. One scenario was a ring-shaped sound source distribution, and the other was a cuboid sound source distribution. These two scenarios represent some specific sound source localization scenarios in practical applications, such as surround sound sources localization and road traffic flow noise sources tracking. For each specific localization scenario, two strategies of structure optimization were adopted to generate two kinds of optimal structures. In addition, the regular polyhedron microphone array structure proposed by Hu et al. as well as random array structures were used as a comparative study of the performance of sound source localization. The model established in Section 1 and Section 2 was edited to code and run on the Matlab platform.

### 4.1. Scenario I—Ring-Shaped Sound Sources Distribution

In scenario I, sound sources were distributed in a cyclic annular band, here referred to as the ring-shaped sound source distribution. The distribution was controlled by Equation (35), as follows:

(35) $$C_{SI}=\left[R_{SI}\sin\left(2\pi \theta_{SI}/360\right),R_{sI}\cos\left(2\pi \theta_{SI}/360\right),h_{SI}\right]$$

where $C_{SI}$ is the coordinates of the sound source. $R_{SI}$ is the radius of the cyclic annular band. $\theta_{SI}$ is the azimuth angle of the source. $h_{SI}$ is the height of the source. In scenario I, $R_{SI}\in \left[6\;m,6.5\;m\right]$, $\theta_{SI}\in \left[0^{\circ },360^{\circ }\right]$, $h_{SI}\in \left[0\;m,0.5\;m\right]$.

**Figure 3:**
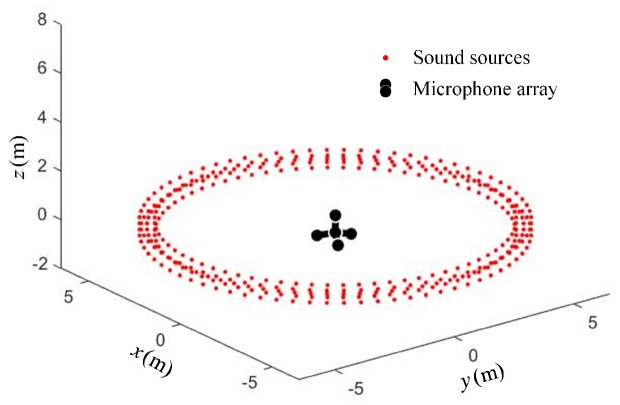
Scenario I—ring-shaped sound source distribution.

The source distribution for the array structure optimization under scenario I is shown in Figure 3. The microphone array was located in the center of the ring, in which the reference microphone was located at the origin of the coordinates, and the location of other positioning microphones was obtained by optimization calculation.

For the sound source localization based on TDOA, the time difference estimation error is the main influencing factor for localization accuracy. To facilitate the optimization and verification of microphone array structures, the TDOAs were directly obtained by calculating the relative position relationship between the sound sources and the array microphones. A noise component was added to the TDOAs, which was used to represent measurement noise in actual applications.

(36) $$r_{i,0}=c\tau_{i,0}+\eta_{i,0},$$

where $\eta_{i,0}$ is the time delay estimation noise component. $\eta_{i,0}$ is assumed to be a mutually independent, zero-mean stationary Gaussian random process, and the standard deviation of $\eta_{i,0}$ is σ. In this simulation, σ was set as 0.01.

Five microphones were selected to optimize the microphone array structures, which were compared with the tetrahedral structure array proposed by Hu et al. as well as a random array. One microphone in the array was chosen as the reference microphone, and the coordinates were set as (0,0,0). The other microphones’ coordinates were set as $M_{i}=\left(l_{i}\cos\left(\beta_{i}\right)\cos\left(\alpha_{i}\right),l_{i}\cos\left(\beta_{i}\right)\sin\left(\alpha_{i}\right),l_{i}\sin\left(\beta_{i}\right)\right)$. The array parameters $\left(l_{i},\alpha_{i},\beta_{i}\right)$ were set as the properties of each particle. The parameters of the PSO model were initialized. The learning factors $c_{1}$ and $c_{2}$ were all set to 1.5. The maximum weight $w_{max}$ was set to 0.8. The minimum weight $w_{min}$ was set to 0.4. The weight value $\phi_{w}$ of the fitness function was set to 0.5, which means that the localization accuracy and robustness were equally considered.

In this simulation, two optimization strategies were applied to search for the optimal array structure. For the first kind of array optimization (Opt-array I), the distances $l_{i}$ between $M_{i}$ and $M_{0}$ were set to the same length 0.7 m, which is comparable with the tetrahedral structure array proposed by Hu et al. and the random array. Then, the azimuth angle $\alpha_{i}$ and the elevation angle $\beta_{i}$ are the main geometric parameters to be optimized for the microphone array. Therefore, for Opt-array I, the dimension of the particles in the PSO algorithm was eight, since the number of other microphones used in scenario I was four. The constraints of the optimization space were $\alpha_{i}\in \left[0^{\circ },360^{\circ }\right]$ and $\beta_{i}\in \left[-90^{\circ },90^{\circ }\right]$. The number of particles *N* was set to 250. For the second kind of array optimization (Opt-array II), the radial distance $l_{i}$ was not predefined. The azimuth angle $\alpha_{i}$, the elevation angle $\beta_{i}$, as well as the radial distances $l_{i}$ were all used for the array geometric parameters to be optimized. For convenience, the radial distance $l_{i}$ was limited between 0.2 m and 0.8 m, considering the general array size for sound source localization. Then, the dimension of the particles for Opt-array II was twelve, and the constraints of the optimization space were $\alpha_{i}\in \left[0^{\circ },360^{\circ }\right]$,$\beta_{i}\in \left[-90^{\circ },90^{\circ }\right]$, and $l_{i}\in \left[0.2\;m,0.8\;m\right]$. Given that the optimizing search space is much larger than in Opt-array I, the number of particles *N* was set to 450 for Opt-array II.

**Figure 4:**
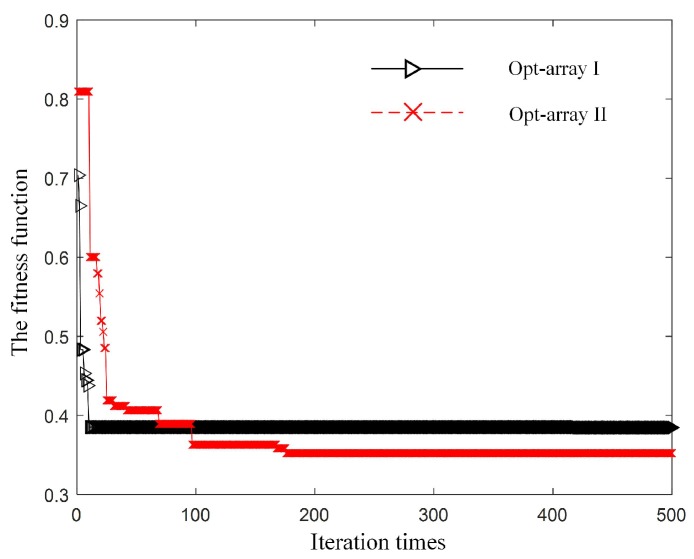
The fitness evolution curve under scenario I for the two kinds of optimal array.

Then, the optimization model based on PSO ran on the Matlab platform to obtain the optimal array structure under scenario I. The fitness evolution curves of Opt-array I and the Opt-array II are shown in Figure 4.

It can be seen from Figure 4 that the fitness evolution curve tended to be stable after 200 steps of iteration, which indicates that the optimization process was basically convergent. The optimization results are deemed to be the optimal array structures under scenario I.

For the manufacturing of a microphone array, an angle precision of $5^{\circ }$ is achievable. Therefore, the angle values of the optimized array were all rounded each $5^{\circ }$. The microphone coordinates and geometric parameters of the optimal arrays, the tetrahedral array proposed by Hu et al., and a random array are listed in Table 1.

**Figure 5:**
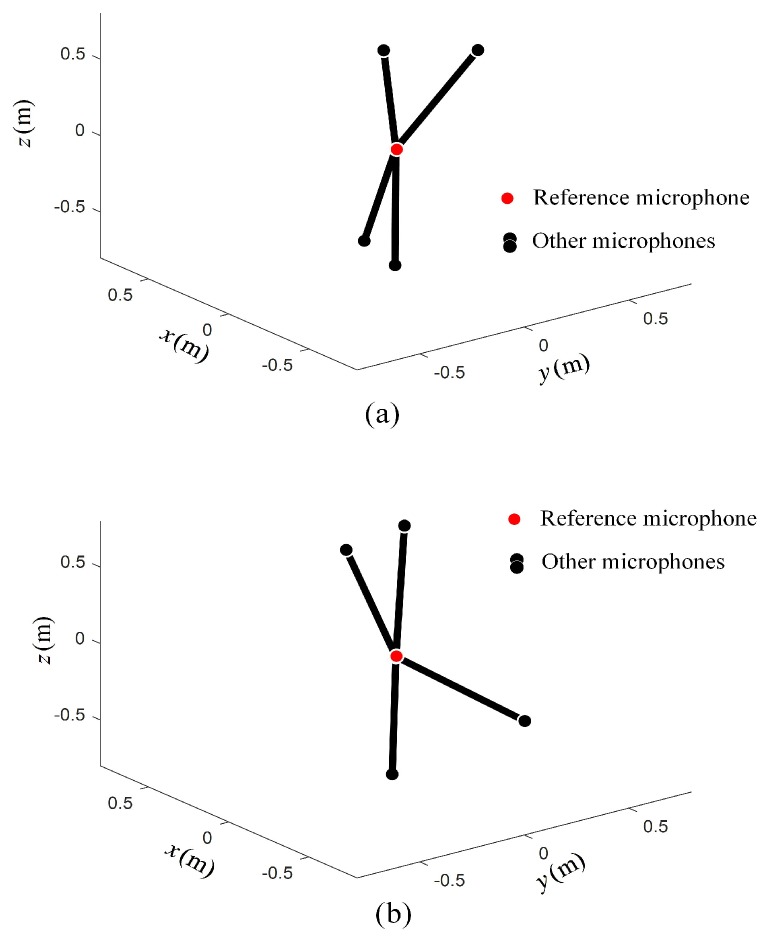
The optimized array structure under scenario I. (**a**) The structure of Opt-array I; (**b**) The structure of Opt-array II.

It can be seen from Table 1 that the array geometric parameters between Opt-array I and the Opt-array II were different. The average radial distances of Opt-array II were larger than those of Opt-array I. At the same time, there were small differences among the four radial distances of Opt-array II. The array structures of Opt-array I and Opt-array II are shown in Figure 5.

**Figure 6:**
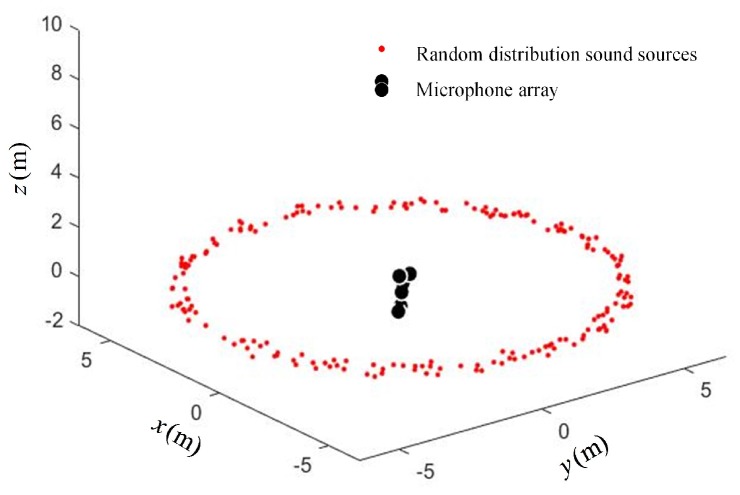
Random distribution of sound sources in the cyclic annular band.

In order to verify the performance of the optimal arrays obtained in the simulation, a scenario with sound sources randomly distributed in the cyclic annular band was constructed, as shown in Figure 6.

**Figure 7:**
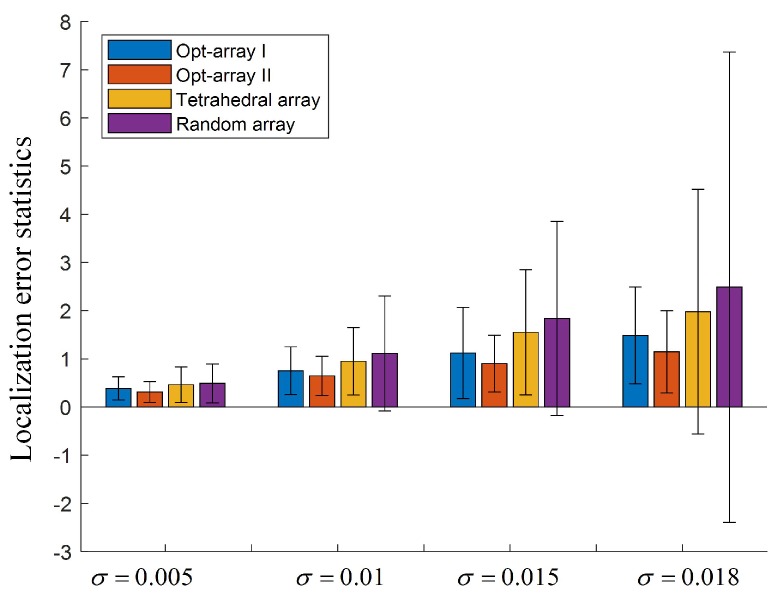
The localization error statistics for the arrays under scenario I.

As shown in Figure 6, 200 sources were randomly distributed in the cyclic annular band of scenario I. Opt-array I, opt-array II, the tetrahedral structure array, and the random array were used to locate these sources. The distances between the located sources and the corresponding real sources were counted to measure the positioning accuracy and robustness. Meanwhile, in order to analyze the influence of input noise on array positioning performance, Gaussian random noises $\eta_{i,0}$ with four different standard deviations were added to the time delay estimation, namely, σ=0.005, σ=0.01, σ=0.015, σ=0.018. The statistical chart is shown in Figure 7.

In Figure 7, the height of the rectangular bar stands for mean localization error, and the length of the line bar presents the standard deviation of the localization error. It can be seen from Figure 7 that the mean values and the standard deviations of the localization error were enlarged with the increase of the input noise component of the time delay estimation. Under the same input noise amplitude, the mean value and the standard deviation of Opt-array I and Opt-array II were all much lower than that of the tetrahedral array and the random array. The bigger the input noise, the more significant the gap. This means that the optimized arrays by the proposed method could improve the accuracy and robustness of the sound source localization based on TDOA. The results illustrate the effectiveness of the array structure optimization method proposed in this paper.

Under four different input noise levels, the mean value and standard deviation of the localization error for the random array were all much larger than that for Opt-array I, Opt-array II, and the tetrahedral array, which illustrates that array optimization—whether the method of this paper or Hu’s method—produced a positive effect. The random arrays may achieve excellent positioning performance, but the possibility is tiny. Also, the mean value and standard deviation of the localization error for Opt-array II were lower than for Opt-array I. Considering that there were differences among the four radial distances $l_{i}$ of Opt-array II after array structure optimization, the optimization of the radial distance $l_{i}$ contributes to promoting the positioning performance of the microphone array besides the optimization of the azimuth angle $\alpha_{i}$ and the elevation angle $\beta_{i}$.

### 4.2. Scenario II—Cuboid-Shaped Sound Sources Distribution

**Figure 8:**
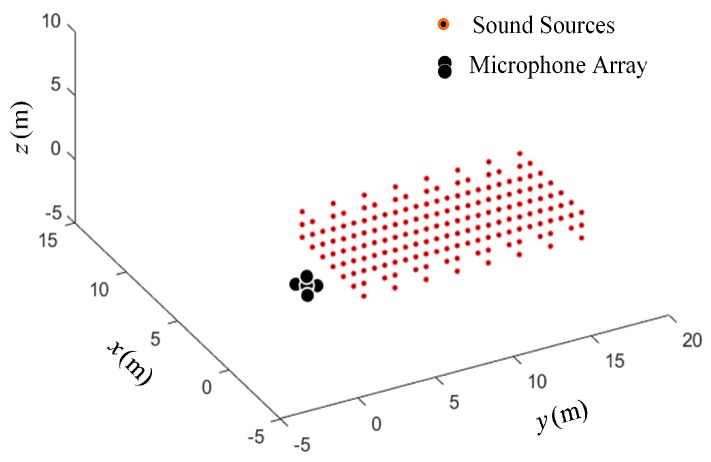
Scenario II–cuboid-shaped sound source distribution.

In scenario II, the sound sources were distributed in a cuboid space band, here referred to as the cuboid-shaped sound source distribution. The cuboid space band is was 15 m × 6 m × 3 m. The microphone array was located on one side of the cuboid distribution. The location of the reference microphone coincided with the origin of the coordinate system. The constructed scenario II is shown in Figure 8.

In the simulation of scenario II, five microphones were also selected to optimize the microphone array structure, which was compared with the tetrahedral structure array proposed by Hu et al. and a random array. The noise component $\eta_{i,0}$ with zero-mean Gaussian normal distribution was introduced into the time delay estimation. The standard deviation σ of the noise was the same as in scenario I, namely, 0.01. The parameters of the PSO model were set to be the same as in scenario I.

**Figure 9:**
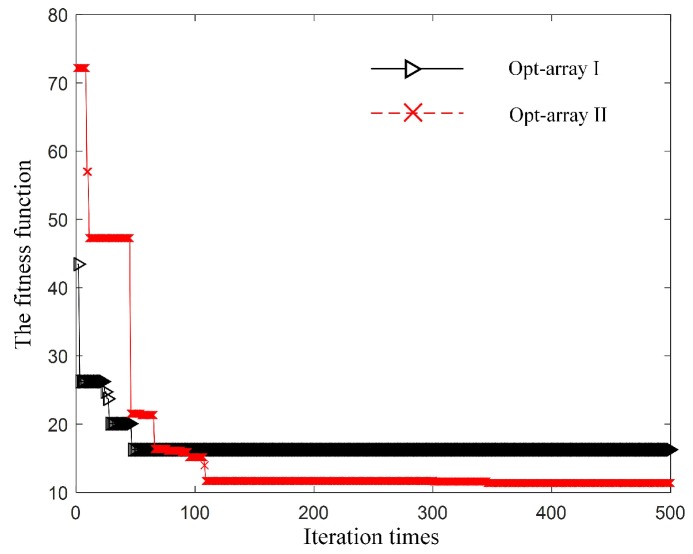
The fitness evolution curve under scenario II for the two kinds of optimal array.

The fitness evolution curve of Opt-array I and Opt-array II under scenario II are shown in Figure 9.

It can be seen from Figure 9 that the fitness evolution curve tended to be stable after 150 steps of iteration. At the beginning of the iteration, the fitness function value of Opt-array II was higher than that of Opt-array I. Nevertheless, after numbers of iterative calculation, the fitness function value of Opt-array II was lower than that of Opt-array I when the iterations approached convergence, which means that the optimized structure of Opt-array II may have better localization performance than Opt-array I. Also, the angle values of the optimized array were all rounded each $5^{\circ }$. The microphone coordinates of the optimal arrays and the tetrahedral array proposed by Hu et al. and the random array are listed in Table 2.

**Figure 10:**
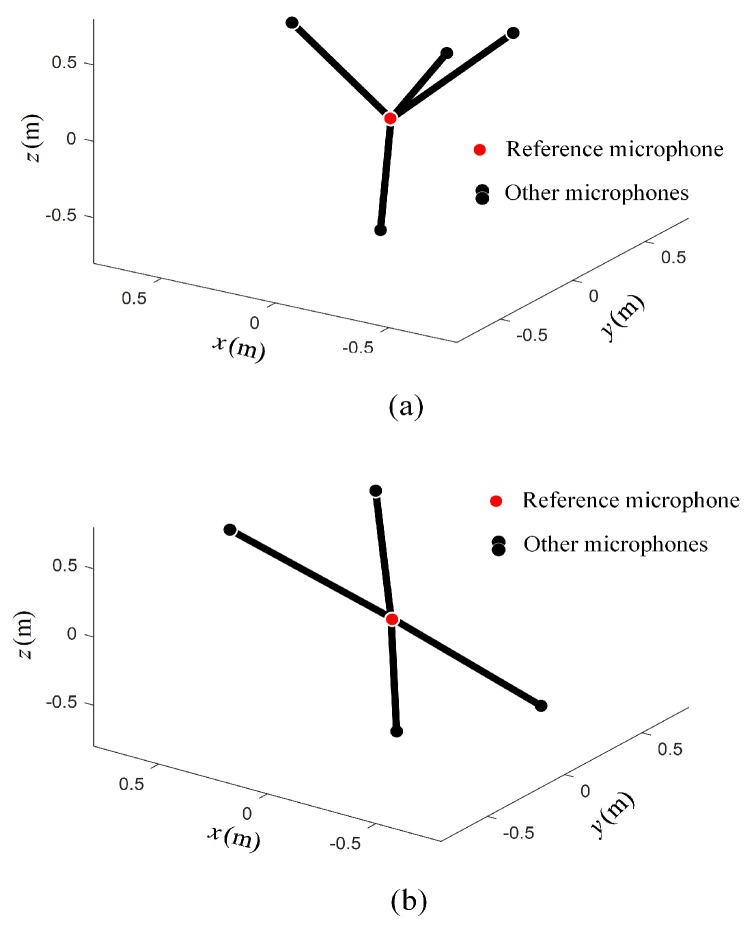
The optimized array structure under scenario II. (**a**) The structure of Opt-array I; (**b**) The structure of Opt-array II.

It can be seen from Table 2 that the array geometric parameters between Opt-array I and Opt-array II under scenario II were different. The difference of radial distances $l_{i}$ of Opt-array II under scenario II was much larger than that under scenario I. The array structures of Opt-array I and Opt-array II are shown in Figure 10.

**Figure 11:**
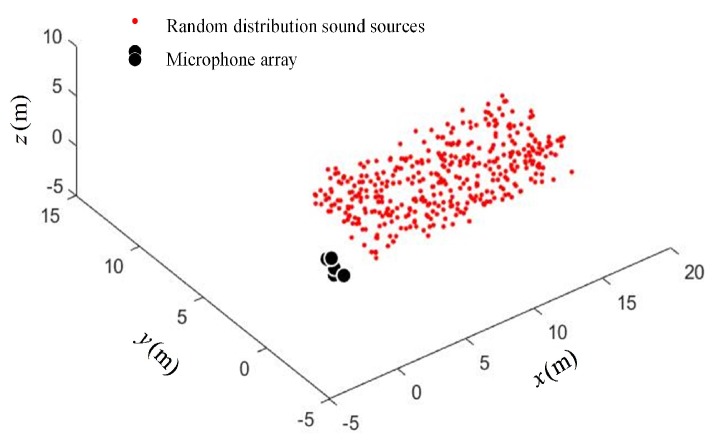
The random distribution of sound sources in the cuboid space band.

In order to verify the performance of the arrays, the scenario of randomly distributed sound sources in the cuboid space band was constructed, as shown in Figure 11.

**Figure 12:**
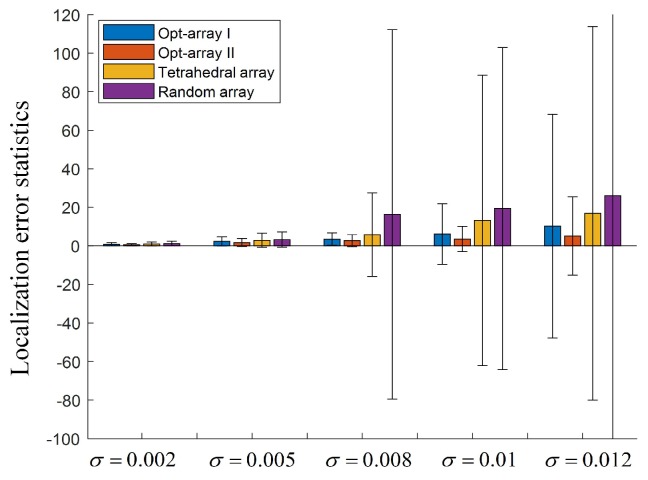
The localization error statistics for the arrays under scenario II.

In Figure 11, 400 sources are randomly distributed in the cuboid space band.The Opt-array I, the Opt-array II, the tetrahedral structure array, and the random array are used to locate these sources. The Gaussian random noise $\eta_{i,0}$ with five different standard deviations are added to the time delay estimation, namely σ=0.002,σ=0.005,σ=0.008,σ=0.01,σ=0.012. The statistics of the distances between the located sources and the corresponding real sources are drawn in Figure 12.

It can be seen from Figure 12 that the mean values and the standard deviations of the localization error were enlarged with the increase of the input noise component of the time delay estimation. Under the same input noise amplitude, the mean value and the standard deviation of Opt-array I and Opt-array II were much lower than those of the tetrahedral array proposed by Hu et al. and the random array, and the gap increased rapidly with the increase of input noise. The optimized arrays by the proposed optimization method could improve the accuracy and robustness of the sound source localization based on TDOA.

Under five different input noise levels, the mean value and standard deviation of the localization error for the random array were much larger than that for Opt-array I, Opt-array II, and the tetrahedral array, especially when the input noise component was large, which illustrates that array optimization produced a positive effect. Random arrays have little chance of achieving excellent positioning performance under specific localization scenarios. Also, the mean value and standard deviation of the localization error for Opt-array II were lower than those for Opt-array I, which indicates that the optimization of the radial distance $l_{i}$ contributed to promoting the positioning performance of the microphone array besides the optimization of the azimuth angle $\alpha_{i}$ and the elevation angle $\beta_{i}$. Moreover, the localization error reduction of Opt-array II under scenario II was more significant than that under scenario I when the input noise component was large. Considering that the difference of radial distance $l_{i}$ of Opt-array II under scenario II was much larger than that under scenario I, the radial distance under scenario II was a more significant factor of the array structure optimization than under scenario I.

In addition, compared with scenario I, the mean values and the standard deviations of scenario II were much larger. The standard deviation rose sharply with the increase of input noise. The main reason for this is that the location area and the size of the sound sources in scenario II were much larger than in scenario I, and the sound sources were asymmetrically distributed. For scenario II, increasing the number of array microphones may help to reduce positioning errors and improve positioning robustness. Therefore, another optimization case was applied in scenario II, which is that seven microphones were chosen for the array structure optimization. The octahedron structure array proposed by Hu et al. [40] and a random array with seven microphones were used for comparative study.

Two kinds of optimization strategies were also used in the simulation. In the first kind of array optimization (Opt-array-7mic I), the radial distances $l_{i}$ between $M_{i}$ and $M_{0}$ were set to the same length of 0.7 m. For the second kind of array optimization (Opt-array-7mic II), the radial distances $l_{i}$, the azimuth angle $\alpha_{i}$, and the elevation angle $\beta_{i}$ were all used as the array geometric parameters to be optimized. The constraints and the initial parameters of the optimization model were set to be the same as in the case of the five microphone array optimization. Given that the optimizing search space was much larger than the array optimization with five microphones, the number of particles *N* for Opt-array-7mic I and Opt-array-7mic II were set to 400 and 650, respectively.

**Figure 13:**
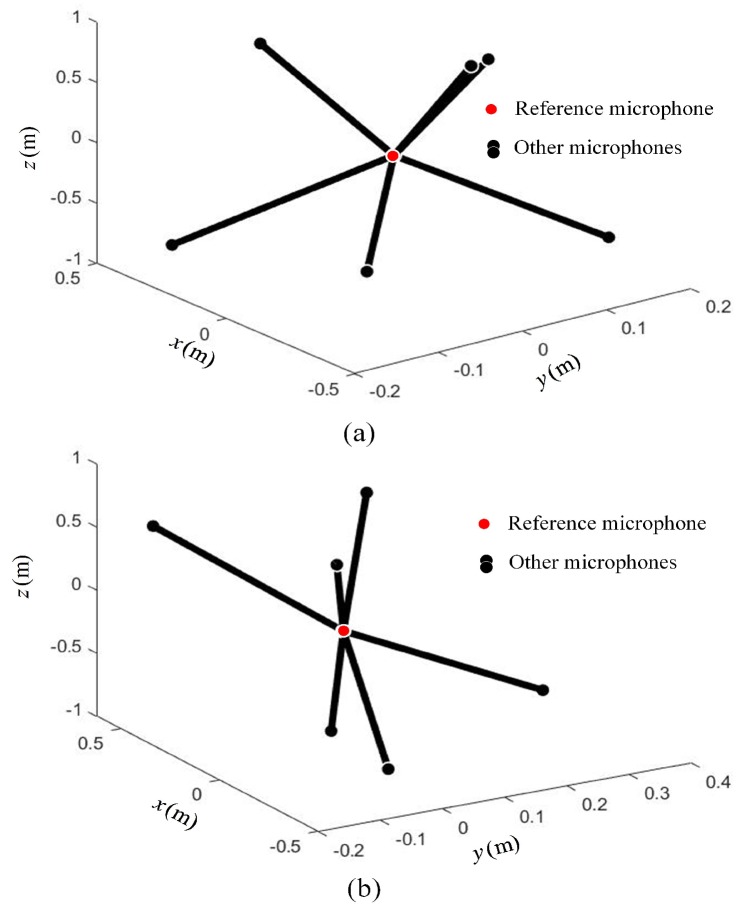
The optimized array structure with seven microphones under scenario II. (**a**) The structure of Opt-array-7mic I; (**b**) The structure of Opt-array-7mic II.

The optimal array structures were obtained after running the optimization model on the Matlab platform under scenario II. The geometric parameters of the optimal arrays, the octahedron array, and the random array are listed in Table 3. Also, the angles of the optimized array were all rounded $5^{\circ }$. The array structures of Opt-array-7mic I and Opt-array-7mic II are shown in Figure 13.

It can be seen from Table 3 and Figure 13 that the array structure between Opt-array-7mic I and Opt-array-7mic II were different. The difference of the radial distances $l_{i}$ of Opt-array-7mic II was much smaller than that of Opt-array II under scenario II.

**Figure 14:**
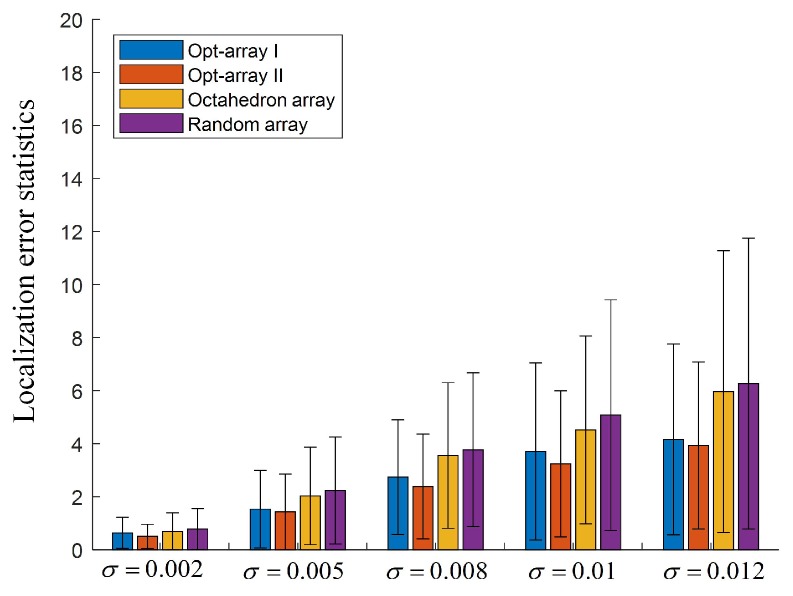
The localization error statistics for the arrays with seven microphones under scenario II.

In order to verify the performance of the optimal arrays, the scenario of randomly distributed sound sources in the cuboid space band was constructed, similar to Figure 11. Gaussian random noises $\eta_{i,0}$ with five standard deviations were also added to the time delay estimation, namely, σ=0.002,σ=0.005,σ=0.008,σ=0.01,σ=0.012. The statistics of the distances between the located sources and the corresponding real sources are drawn in Figure 14.

Figure 14 shows that the mean values and standard deviations of localization error for Opt-array-7mic I and Opt-array-7mic II were lower than for the octahedron array proposed by Hu et al. and the random array, which illustrates the effectiveness of the proposed array optimization method. Comparing Figure 12 and Figure 14, it can be seen that the mean values and the standard deviations of localization error for Opt-array-7mic I and Opt-array-7mic II were lower than for Opt-array-I and Opt-array II under scenario II. Considering that the standard deviations of the optimal arrays with seven microphones were significantly lower than those of the optimal arrays with five microphones when the input noise component was large, optimal array structures with more microphones could significantly improve the robustness of the source localization based on TDOA. In addition, the mean values and the standard deviations of localization error for the octahedron array and the random array with seven microphones were also much lower than that for the tetrahedral array and the random array with five microphones, which demonstrates that increasing the number of microphones can greatly improve the positioning accuracy and robustness of the array based on TDOA.

## 5. Conclusions

This paper proposed a method of microphone array optimization for sound source localization based on TDOA under specific localization scenarios, which can be applied to the optimization of arbitrary array structure without prior information. For any number of microphones, a more optimal array structure can be given under any localization scenario. The proposed method is a numerical approach based on the particle swarm optimization algorithm. The mean squared error and the variance of the localization results combined with a weight value are used to construct the fitness function of the optimization model, which can consider both positioning accuracy and robustness. The geometric structure of the microphone array was established in parametric form, which is assigned as particle attributes and substituted into the optimization model to obtain the more optimal results. Two specific localization scenarios were constructed to optimize the array structures. For both specific scenarios, two kinds of array optimization strategies were utilized to obtain two optimal array structures. The optimized array structures were compared with the regular polyhedron structure array under different input noise amplitude.

For scenario I, the mean value and the standard deviation of the localization error for Opt-array I and Opt-array II were much lower than for the tetrahedral array and the random array, and the higher the input noise, the more significant the gap. Under four different input noise levels, the mean value and standard deviation of the localization error for the random array were the largest, and those of Opt-array II were the smallest. The results indicate that the array optimization produced a positive effect, and the optimization of the radial distance $l_{i}$ contributed to promoting the positioning performance of the microphone array under scenario I.

For scenario II, the mean value and the standard deviation of Opt-array I and Opt-array II were also much lower than those of the tetrahedral array and the random array. Under five different input noise levels, the mean value and standard deviation of the localization error for the random array were the largest, and those of Opt-array II were the smallest. The array optimization and the optimization of the radial distance $l_{i}$ all showed a positive effect on the positioning performance of the microphone array under scenario II. Moreover, the localization error reduction of Opt-array II under scenario II was more significant than that under scenario I. Considering that the difference of the radial distance of Opt-array II under scenario II was much larger than that under scenario I, the radial distance under scenario II was a more significant factor of the array structure optimization than that under scenario I. Under scenario II, the mean value and standard deviation of the optimal array were much higher than those of the optimal array under scenario I. The array with seven microphones was introduced into the optimization under scenario II, compared with the octahedron array and a random array. The results show that under five different input noise levels, the mean value and standard deviation of the localization error for Opt-array-7mic II were the smallest, and those for the random array were the largest. The mean value and standard deviation of the optimal array with seven microphones were lower than those of the optimal array with five microphones; especially, the standard deviation of the optimal array was significantly lower. This indicates that an optimal array structure with more microphones can significantly improve the robustness of source localization based on TDOA.

For both specific localization scenarios, the comparison results show that the localization accuracy and robustness of the optimized array structures were better than those of the regular polyhedron array structures proposed by Hu et al. and random array structures, which illustrates the effectiveness of the proposed array structure optimization method. The random arrays may achieve excellent positioning performance, but the likelihood is small. The optimization of the radial distance $l_{i}$ contributed to promoting the positioning performance of the microphone array besides the optimization of the azimuth angle $\alpha_{i}$ and the elevation angle $\beta_{i}$, particularly for scenario II. In the future, the efficiency of the optimization algorithm can be further studied, as well as the correlation between the positioning performance of the array and the array geometric parameters.

## Figures and Tables

**Table 1:** The microphone coordinates of the arrays under scenario I.

Items	Opt-Array I	Opt-Array II	Tetrahedral Array	Random Array
Coordinates	$M_{0}\left(0,0,0\right)$	$M_{0}\left(0,0,0\right)$	$M_{0}\left(0,0,0\right)$	$M_{0}\left(0,0,0\right)$
	$M_{1}\left(-0.09,-0.04,0.69\right)$	$M_{1}\left(0.10,0.07,0.79\right)$	$M_{1}\left(0,0,0.7\right)$	$M_{1}\left(0.56,0.32,0.38\right)$
	$M_{2}\left(-0.08,-0.1,-0.69\right)$	$M_{2}\left(0.24,-0.49,-0.28\right)$	$M_{2}\left(0.66,0,-0.24\right)$	$M_{2}\left(0,-0.54,0.54\right)$
	$M_{3}\left(0.27,-0.16,0.63\right)$	$M_{3}\left(0.01,0.04,-0.79\right)$	$M_{3}\left(-0.33,0.57,-0.24\right)$	$M_{3}\left(-0.58,-0.21,-0.29\right)$
	$M_{4}\left(-0.02,0.18,-0.68\right)$	$M_{4}\left(-0.22,0.02,0.77\right)$	$M_{4}\left(-0.33,-0.57,-0.24\right)$	$M_{4}\left(0.06,-0.11,-0.71\right)$
Array Geometric Parameters	$l_{i}=0.7\;m$	$l_{1}=0.75\;m$	$l_{i}=0.7\;m$	$l_{1}=0.75\;m$
		$l_{2}=0.73\;m$		$l_{2}=0.77\;m$
		$l_{3}=0.77\;m$		$l_{3}=0.68\;m$
		$l_{4}=0.75\;m$		$l_{4}=0.72\;m$
	$\alpha_{1}=25^{\circ }$; $\beta_{1}=100^{\circ }$	$\alpha_{1}=35^{\circ }$; $\beta_{1}=80^{\circ }$	$\alpha_{1}=0^{\circ }$; $\beta_{1}=90^{\circ }$	$\alpha_{1}=30^{\circ }$; $\beta_{1}=30^{\circ }$
	$\alpha_{2}=230^{\circ }$; $\beta_{2}=-80^{\circ }$	$\alpha_{2}=295^{\circ }$; $\beta_{2}=-25^{\circ }$	$\alpha_{2}=0^{\circ }$; $\beta_{2}=-20^{\circ }$	$\alpha_{2}=270^{\circ }$; $\beta_{2}=45^{\circ }$
	$\alpha_{3}=330^{\circ }$; $\beta_{3}=65^{\circ }$	$\alpha_{3}=75^{\circ }$; $\beta_{3}=-85^{\circ }$	$\alpha_{3}=120^{\circ }$; $\beta_{3}=-20^{\circ }$	$\alpha_{3}=200^{\circ }$; $\beta_{3}=-25^{\circ }$
	$\alpha_{4}=95^{\circ }$; $\beta_{4}=-75^{\circ }$	$\alpha_{4}=175^{\circ }$; $\beta_{4}=75^{\circ }$	$\alpha_{4}=240^{\circ }$; $\beta_{4}=-20^{\circ }$	$\alpha_{4}=300^{\circ }$; $\beta_{4}=-80^{\circ }$

**Table 2:** The microphone coordinates of the arrays under scenario II.

Items	Opt-Array I	Opt-Array II	Tetrahedral Array	Random Array
Coordinates	$M_{0}\left(0,0,0\right)$	$M_{0}\left(0,0,0\right)$	$M_{0}\left(0,0,0\right)$	$M_{0}\left(0,0,0\right)$
	$M_{1}\left(0.29,-0.34,0.54\right)$	$M_{1}\left(-0.26,-0.22,-0.58\right)$	$M_{1}\left(0,0,0.7\right)$	$M_{1}\left(0.56,0.32,0.38\right)$
	$M_{2}\left(-0.14,0.32,0.61\right)$	$M_{2}\left(0.17,-0.57,-0.50\right)$	$M_{2}\left(0.66,0,-0.24\right)$	$M_{2}\left(0,-0.54,0.54\right)$
	$M_{3}\left(-0.06,0.01,-0.70\right)$	$M_{3}\left(0.13,0.16,0.78\right)$	$M_{3}\left(-0.33,0.57,-0.24\right)$	$M_{3}\left(-0.58,-0.21,-0.29\right)$
	$M_{4}\left(0.68,0.18,0\right)$	$M_{4}\left(-0.12,0.67,0.40\right)$	$M_{4}\left(-0.33,-0.57,-0.24\right)$	$M_{4}\left(0.06,-0.11,-0.71\right)$
Array Geometric Parameters	$l_{i}=0.7\;m$	$l_{1}=0.73\;m$	$l_{i}=0.7\;m$	$l_{1}=0.75\;m$
		$l_{2}=0.65\;m$		$l_{2}=0.77\;m$
		$l_{3}=0.80\;m$		$l_{3}=0.68\;m$
		$l_{4}=0.69\;m$		$l_{4}=0.72\;m$
	$\alpha_{1}=130^{\circ }$; $\beta_{1}=130^{\circ }$	$\alpha_{1}=220^{\circ }$; $\beta_{1}=-60^{\circ }$	$\alpha_{1}=0^{\circ }$; $\beta_{1}=90^{\circ }$	$\alpha_{1}=30^{\circ }$; $\beta_{1}=30^{\circ }$
	$\alpha_{2}=115^{\circ }$; $\beta_{2}=60^{\circ }$	$\alpha_{2}=285^{\circ }$; $\beta_{2}=-40^{\circ }$	$\alpha_{2}=0^{\circ }$; $\beta_{2}=-20^{\circ }$	$\alpha_{2}=270^{\circ }$; $\beta_{2}=45^{\circ }$
	$\alpha_{3}=175^{\circ }$; $\beta_{3}=-85^{\circ }$	$\alpha_{3}=50^{\circ }$; $\beta_{3}=75^{\circ }$	$\alpha_{3}=120^{\circ }$; $\beta_{3}=-20^{\circ }$	$\alpha_{3}=200^{\circ }$; $\beta_{3}=-25^{\circ }$
	$\alpha_{4}=15^{\circ }$; $\beta_{4}=0^{\circ }$	$\alpha_{4}=100^{\circ }$; $\beta_{4}=30^{\circ }$	$\alpha_{4}=240^{\circ }$; $\beta_{4}=-20^{\circ }$	$\alpha_{4}=300^{\circ }$; $\beta_{4}=-80^{\circ }$

**Table 3:** The geometric parameters of the arrays with 7 microphones under the scenario II.

Items	Opt-Array-7mic I	Opt-Array-7mic II	Octahedron Array	Random Array
Coordinates	$M_{0}\left(0,0,0\right)$	$M_{0}\left(0,0,0\right)$	$M_{0}\left(0,0,0\right)$	$M_{0}\left(0,0,0\right)$
	$M_{1}\left(0.10,-0.07,0.69\right)$	$M_{1}\left(0.33,-0.03,-0.72\right)$	$M_{1}\left(0,0,0.7\right)$	$M_{1}\left(0.56,0.32,0.38\right)$
	$M_{2}\left(-0.08,-0.16,-0.68\right)$	$M_{2}\left(-0.17,0.47,0.60\right)$	$M_{2}\left(-0.495,-0.495,0\right)$	$M_{2}\left(0,-0.54,0.54\right)$
	$M_{3}\left(0,0.50,0.50\right)$	$M_{3}\left(0.23,0.62,0.38\right)$	$M_{3}\left(0.495,-0.495,0\right)$	$M_{3}\left(-0.58,-0.21,-0.29\right)$
	$M_{4}\left(0.14,-0.38,-0.57\right)$	$M_{4}\left(-0.09,-0.50,-0.61\right)$	$M_{4}\left(0.495,0.495,0\right)$	$M_{4}\left(0.06,-0.11,-0.71\right)$
	$M_{5}\left(0.08,-0.09,0.69\right)$	$M_{5}\left(-0.05,-0.05,-0.75\right)$	$M_{5}\left(-0.495,0.495,0\right)$	$M_{5}\left(0.79,0,0\right)$
	$M_{6}\left(-0.18,0.23,-0.63\right)$	$M_{6}\left(-0.07,-0.19,0.75\right)$	$M_{6}\left(0,0,-0.7\right)$	$M_{6}\left(-0.18,0.30,0.61\right)$
Array Geometric Parameters	$l_{i}=0.7\;m$	$l_{1}=0.79\;m$	$l_{i}=0.7\;m$	$l_{1}=0.75\;m$
		$l_{2}=0.78\;m$		$l_{2}=0.77\;m$
		$l_{3}=0.76\;m$		$l_{3}=0.68\;m$
		$l_{4}=0.79\;m$		$l_{4}=0.72\;m$
		$l_{5}=0.75\;m$		$l_{5}=0.79\;m$
		$l_{6}=0.78\;m$		$l_{6}=0.70\;m$
	$\alpha_{1}=325^{\circ }$; $\beta_{1}=80^{\circ }$	$\alpha_{1}=355^{\circ }$; $\beta_{1}=-65^{\circ }$	$\alpha_{1}=0^{\circ }$; $\beta_{1}=90^{\circ }$	$\alpha_{1}=30^{\circ }$; $\beta_{1}=30^{\circ }$
	$\alpha_{2}=245^{\circ }$; $\beta_{2}=-75^{\circ }$	$\alpha_{2}=110^{\circ }$; $\beta_{2}=50^{\circ }$	$\alpha_{2}=225^{\circ }$; $\beta_{2}=0^{\circ }$	$\alpha_{2}=270^{\circ }$; $\beta_{2}=45^{\circ }$
	$\alpha_{3}=90^{\circ }$; $\beta_{3}=45^{\circ }$	$\alpha_{3}=70^{\circ }$; $\beta_{3}=30^{\circ }$	$\alpha_{3}=315^{\circ }$; $\beta_{3}=0^{\circ }$	$\alpha_{3}=200^{\circ }$; $\beta_{3}=-25^{\circ }$
	$\alpha_{4}=290^{\circ }$; $\beta_{4}=-55^{\circ }$	$\alpha_{4}=260^{\circ }$; $\beta_{4}=-50^{\circ }$	$\alpha_{4}=45^{\circ }$; $\beta_{4}=0^{\circ }$	$\alpha_{4}=300^{\circ }$; $\beta_{4}=-80^{\circ }$
	$\alpha_{5}=310^{\circ }$; $\beta_{5}=80^{\circ }$	$\alpha_{5}=225^{\circ }$; $\beta_{5}=-85^{\circ }$	$\alpha_{5}=135^{\circ }$; $\beta_{5}=0^{\circ }$	$\alpha_{5}=0^{\circ }$; $\beta_{5}=0^{\circ }$
	$\alpha_{6}=130^{\circ }$; $\beta_{6}=-65^{\circ }$	$\alpha_{6}=250^{\circ }$; $\beta_{6}=75^{\circ }$	$\alpha_{6}=0^{\circ }$; $\beta_{6}=-90^{\circ }$	$\alpha_{6}=120^{\circ }$; $\beta_{6}=60^{\circ }$

## Abbreviations

The following abbreviations are used in this manuscript:

TDOA	Time Difference of Arrival
PSO	Particle Swarm Optimization
